# Variations in stakeholders' priorities and views on randomisation and funding decisions in out‐of‐hospital cardiac arrest: An exploratory study

**DOI:** 10.1002/hsr2.78

**Published:** 2018-07-25

**Authors:** Johannes von Vopelius‐Feldt, Janet Brandling, Jonathan Benger

**Affiliations:** ^1^ Academic Department of Emergency Care University Hospitals Bristol NHS Foundation Trust Bristol UK; ^2^ Emergency and Critical Care Research, Faculty of Health & Applied Sciences University of the West of England Bristol UK

**Keywords:** air ambulances, charities, critical care, emergency medical services, focus groups, out‐of‐hospital cardiac arrest, patient participation, research personnel, resource allocation

## Abstract

**Background and aims:**

Prehospital critical care for out‐of‐hospital cardiac arrest (OHCA) is a complex and largely unproven intervention. During research to examine this intervention, we noted significant differences in stakeholders' views about research, randomisation, and the funding of prehospital critical care for OHCA. We aimed to answer the following questions: What are stakeholders' priorities for prehospital research? What are stakeholders' views on randomisation of prehospital critical care? How do stakeholders consider allocation of resources in prehospital care?

**Methods:**

We undertook an explanatory qualitative framework analysis of interviews and focus group with 5 key stakeholder groups: patients and public, air ambulance charities, ambulance service commissioners, prehospital researchers, and prehospital critical care providers.

**Results:**

We undertook 3 focus group discussions with a total of 23 participants and 8 interviews with a total of 9 participants. Despite sharing a common appreciation of the concepts of scientific enquiry, fairness, and beneficence, the 5 relevant stakeholder groups displayed divergent views of research and funding strategies regarding the intervention of prehospital critical care for the condition of OHCA. The reasons for this divergence could largely be explained through the different personal experiences and situational contexts of each stakeholder group. Many aspects of the strategies suggested by the stakeholder groups only partially aligned with principles of traditional evidence‐based medicine, but were held with strong conviction.

**Discussion:**

Analysis of the views of 5 stakeholder groups regarding research and the funding of prehospital critical care for OHCA revealed shared values but a variety of different strategies to achieve these. This knowledge can help researchers in similar fields in the planning and presentation of their research, to maximise impact on decision making.

## 1 INTRODUCTION

Out‐of‐hospital cardiac arrest (OHCA) is defined as the sudden cessation of cardiac activity, leading to collapse and absence of signs of life, outside of the hospital setting.^1^ It is frequently caused by underlying ischaemic heart disease and considered to be 1 of the main causes of mortality worldwide.^2^ In the UK, the incidence of OHCA is estimated at over 28 000 per year, with a survival rate of less than 10%.^3^ The current standard treatment entails advanced life support (ALS), consisting of lung ventilation with oxygen, CPR, defibrillation, and administration of intravenous medication.^4^ In an effort to improve outcomes, some regions in the UK dispatch prehospital critical care teams to OHCAs, in addition to the standard of ALS paramedic care.^5^ Prehospital critical care can be described as a bundle of interventions beyond the remit of standard emergency medical service (EMS) treatment, delivered by a group of specialist prehospital health care providers.^6^ The mechanisms by which prehospital critical care might improve outcomes in OHCA are the experience of the providers, advanced interventions (such as prehospital anaesthesia), or the ability to transfer patients over greater distances to cardiac arrest centres, using equipment and interventions not available to ALS paramedics.^6^ However, there is currently no clear evidence that prehospital critical care improves survival following OHCA when compared to ALS care.^5^ Prehospital critical care is funded through a complex and variable combination of charity support (particularly if associated with an air ambulance) and National Health Service (NHS) ambulance service funding.^7^, ^8^ Researching potential benefits from prehospital critical care for OHCA is important to guide further funding, but is challenging because of the complexity of prehospital critical care itself and the limitations of undertaking prehospital research in OHCA. The Medical Research Council recommends researchers evaluating complex interventions to “*involve stakeholders in the choice of question and design of the research to ensure relevance*”.^9^ Furthermore, the document encourages researchers to “*always consider randomisation, because it is the most robust method of preventing […] selection bias*”.^9^ During the planning process for a research project to investigate prehospital critical care for OHCA, we discussed whether this should be a randomised or observational study design, with an OHCA patient and public involvement (PPI) group, other researchers, and clinical colleagues working in EMS. The different stakeholder groups disagreed strongly about the ethical acceptability of randomising the intervention of prehospital critical care for OHCA, the information that is required to direct health care funding, and even the need to research the question at all. Given the importance of undertaking stakeholder‐relevant research, we decided to formally investigate this wide range of relevant stakeholder views. Using prehospital critical care for OHCA as an example of a complex intervention, this qualitative research aims to answer the following questions:

- What are stakeholders' priorities for prehospital research?
- What are stakeholders' views on randomisation of prehospital critical care, and what are the underlying principles?
- How do stakeholders consider allocation of resources in prehospital care?

We hope that an awareness and understanding of the differences in stakeholders' views can improve the dialogue between stakeholders and help in future planning of research of complex interventions, particularly in prehospital care. The following paragraph provides a short overview of relevant factors of the intervention and condition discussed in this research.

## 2 METHODS

This qualitative research used a pragmatic mixture of stakeholder focus groups and interviews, followed by framework analysis to address the research questions listed in the introduction. The focus is on providing useful and applicable information for all stakeholders involved in prehospital care research. We initially planned for data from all stakeholder groups to be collected through focus groups. However, because of the geographic dispersion and limited availability of some of the participants, for 2 out of the 5 stakeholder groups (air ambulance charity staff and prehospital researchers), this had to be changed to interviews. The research team monitored for signs of bias which might be introduced through this mixture of data collection methods, and the conduct of the interviews closely mirrored that of the focus groups.

### 2.1 Research paradigm

The researcher team consisted of 3 members. JVVF and JB work as emergency medicine and prehospital physicians, and have published largely quantitative research with an underlying positivist epistemology.^10^, ^11^ JBR is a research fellow who has focused on qualitative research in a variety of health care settings^12^ and has previously worked with JB on a qualitative study in prehospital care.^13^ This exploration of stakeholders' views of prehospital critical care for OHCA is nested within a quantitative analysis of the effects of this complex intervention on survival after OHCA. The limitations and challenges of research in prehospital care and particularly OHCA require a certain degree of flexibility to the methods used.^14^ We, therefore, adopted a pragmatic research paradigm, which is reflected in the mixed‐methods structure of the overarching project.^15^, ^16^ Research paradigms can be seen as the link between the aim and the methods of a research project, and described by their ontology, epistemology, and methodology.^17^ To the research presented here, under the pragmatic paradigm, reality is considered to be something to be negotiated and agreed on (ontology).^18^ The pragmatic paradigm does not prescribe a particular epistemology or methodology but considers approaches valid if they work and provide useful results.^18^ With this focus on useful results in mind, we chose the framework approach for data analysis, which falls under the wider method of thematic analysis.^19^

### 2.2 Participant selection

We identified stakeholder groups for a hypothetical randomised controlled trial of prehospital critical care for OHCA, based on the authors' experience in previous relevant research and in preparing the fellowship application for this project.^10^, ^15^

The groups varied significantly in their background, availability to attend focus groups or interviews, and their geographic distribution. The recruitment process was, therefore, tailored to each group to achieve effective recruitment. A description of the recruitment method for each group can be found in Appendix A. For each group, we aimed for 4 to 10 participants in the focus groups and 4 to 6 participants in interviews. To allow stakeholders to comfortably express their opinions and to fully explore each stakeholder group's view, we chose to undertake data collection in homogenous groups, rather than mixing participants from different stakeholder groups in the same focus group. This decision was based on the strength of opinions and emotive reactions of stakeholders encountered by the authors during the preparation phase.

### 2.3 Ethics and consent

The study was reviewed and approved by the Sheffield National Research Ethics Service Committee, York and Humber on July 29, 2016, reference number 16/YH/0300. All participants were given written patient information sheets, and written consent was obtained prior to the focus groups/interviews.

### 2.4 Conduct of the focus groups/interviews

All selected stakeholder groups were known to have previous experience in and/or an understanding of prehospital research. Prior to each focus group/ interview, participants were given a short presentation on the overarching research project and the issues outlined in the introduction section (see Appendix B). Participants were asked if they required any further information prior to starting. This was only requested by the PPI group who considered a clear understanding of the difference between the 2 potential interventions (ALS and prehospital critical care) to be essential for the discussion. If confusion or misunderstandings arose, particularly to the difference between randomised controlled and observational research, this was explained during the focus group/interview, where needed. The duration of the focus group discussions was 45 to 90 minutes; the interviews lasted 30 to 60 minutes. All focus group discussions and interviews were undertaken in a semistructured fashion.^20^ The same question guide was used for focus group discussions and interviews and was constructed to explore the 3 key questions underpinning this research, as outlined in the introduction section (research priorities, randomisation, and funding decisions; see Appendix B). Each subject was introduced with an open question; follow‐up questions were increasingly directed as required. Only minimal refinement of the question guide was required over the course of the research, as well as minor adjustments to accommodate each stakeholder groups' distinct background. All focus groups and interviews were undertaken by JVVF and audio recorded. In addition, JVVF took brief field notes during the interviews/discussions. Because of budget limitations and the logistics of undertaking interviews in various locations, we were unable to provide a second person to facilitate the focus groups/interviews. To minimise potential bias from this limitation, JBR debriefed JVVF after each focus group and after completion of interviews for each stakeholder group. The debriefs also included monitoring for any effects of the data collection method. Key differences between the 2 methods are that focus groups allow for interaction between participants, while interviews ensure that each participant can fully express their opinions.^16^, ^20^ All recordings were transcribed using a professional transcription service.

### 2.5 Data analysis

The field notes, recordings, and transcripts were analysed using a framework approach.^19^ JVVF undertook the analysis, with support from JBR who reviewed the findings regularly. In addition, we received independent feedback on data analysis from a lay person outside the study team who was a member of the transcription service. Analysis followed a 5‐step approach and was undertaken using N‐Vivo software (version 11). The 5 steps were as follows:

1. *Coding*. We reviewed all transcripts multiple times. We used a mixture of predefined codes (deductive element), based on our previous experiences, and combined these with an open coding strategy (inductive element) to include possible unexpected but important themes.
2. *Construction of a thematic framework*. All codes were reviewed and arranged according to the 3 predetermined topics (research priorities, attitudes towards randomisation, and funding strategies). Within each of the 3 topics, codes were grouped into themes and subthemes which emerged during the analysis, creating an initial framework.
3. *Indexing*. The framework created in step 2 was systematically applied to all transcripts, while paying particular attention to any data that might not fit the framework.
4. *Charting*. Data supporting the themes and subthemes was condensed and rearranged within the framework to facilitate analysis. For each topic, this was done first by case (stakeholder group), then by theme.
5. *Mapping and interpretation*. We mapped the range and nature of themes as well as their interactions and relationships. We searched for underlying structures and explanations for the findings of the framework.

To data saturation, there is only limited data to base an accurate estimation on, as some of the stakeholder groups have, to our knowledge, never been researched. Given the anticipated homogeneity of views within each stakeholder group, we anticipated that views could be explored sufficiently within 1 focus group or 4 interviews for each stakeholder group. JVVF and JBR assessed whether the discussions were exhausted and/or views fully explored after each focus group/4 stakeholder group interviews. If we considered further focus groups or interviews with a given stakeholder group to be of potential benefit, the protocol allowed for a further round of focus groups and/or another 4 interviews per stakeholder group. The decision on whether to extend data collection in this fashion was based on a consensus between JVVF and JBR, rather than predetermined criteria.

### 2.6 Data presentation

In keeping with the research questions, we will present the results according to the 3 main topics.

- Topic 1: priorities influencing prehospital research
- Topic 2: randomisation of prehospital critical care for OHCA
- Topic 3: funding decision making

After demonstrating the contrasts between the stakeholder groups' views, the discussion section will aim to identify common underlying values through the application of concepts identified in the literature (fairness, beneficence, and scientific enquiry).

## 3 RESULTS

In total, 23 people participated in 3 focus group discussions and 9 people participated in 8 interviews (1 interview with air ambulance charity representatives included 2 members of the same charity). See Table 1 for an overview of the demographics of the participants, according to stakeholder group.

**Table 1:** Participant demographics according to stakeholder group

Stakeholder Group	Format (Location)	Participants
Patient and public involvement	Focus group (hospital meeting room)	Four female and 5 male Age 50s to 70s Eight participants were survivors, close friends, or direct relatives of survivors of OHCA. One participant survived a cardiac arrest in hospital. Issues relating to prehospital research, including ethics, have been discussed previously in this group.
Air ambulance charities	Interviews (respective charity's office)	Three female and 2 male Age 40s to 60s All participants were senior nonclinical staff of UK air ambulance charities. All charities represented had been involved in prehospital research before.
Ambulance service commissioners	Focus group (commissioners' meeting room)	Four female and 2 male Age 40s to 60s All participants were part of a national ambulance service commissioning group and regularly discuss the funding of prehospital care.
Prehospital researchers	Interviews (individual participant's office)	Two female and 2 male Age 30s to 60s All participants had published prehospital research. Academic experience ranged from junior (PhD applicant) to senior (university professor) researcher.
Prehospital provider	Focus group (local ambulance station meeting room)	Eight male, no female Age 20s to 50s All participants were members of the same critical care service. The group meets every few months for training and to discuss clinical issues.

### 3.1 Topic 1: priorities influencing prehospital research.

#### 3.1.1 Main themes

- Broad support for research to improve prehospital care
- Differences in stakeholders' strategies to improve prehospital care

**Figure 1:**
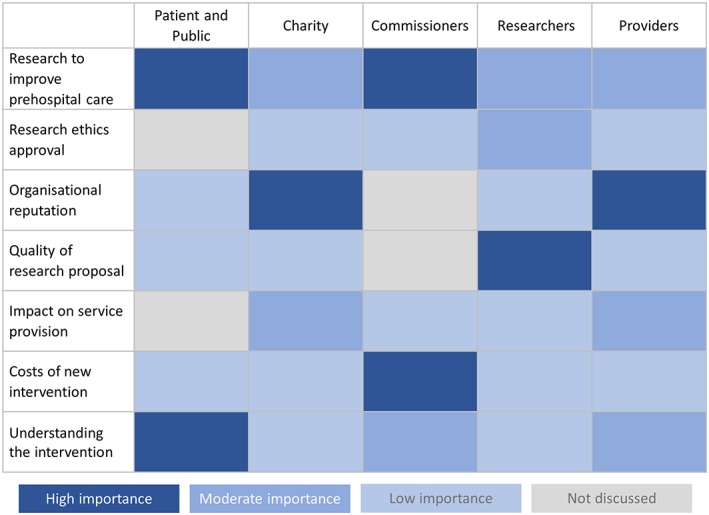
Visual representation of the priorities influencing prehospital research, according to stakeholder group

As hypothesised, views regarding the priorities influencing prehospital research were similar within each stakeholder groups but differed significantly between groups. Figure 1 provides an overview of the importance of the main priorities for each stakeholder group based on the frequency that the topic occurred, the strength of the opinions expressed (as judged by the research team during data analysis), and whether participants discussed the topic spontaneously or after prompting.

The main theme which emerged from the discussion of priorities influencing research was the consistent emphasis by all stakeholders on their support for prehospital research with the aim of improving prehospital care. While all groups agreed on the importance of improvements in prehospital care, each stakeholder group differed in their approach to this priority. Table 2 illustrates how strategies to improve prehospital care through research are determined by the context and perspective of each stakeholder group. See Table 3 for representative quotes from all stakeholder groups.

**Table 2:** Context, perspective, and resulting strategies of stakeholder groups

Stakeholder Group	Context	Perspective	Strategy to Improve Prehospital Care
PPI	Personal experience of life‐threatening, dramatic event and interventions	Individual Patients with OHCA	Optimise care for every patient with OHCA
Charities	Relying on public funding to provide prehospital care additional to NHS care	Regional Patients with severe injury or illness	Support and optimise air ambulance practice
Commissioners	Constrained budget, with decision‐making limited by guidelines and policy	National All patients receiving prehospital care	Maximise benefits to all patients, within budget limits
Researchers	Discuss and analyse questions in theoretical/hypothetical terms	International All patients	Create high‐quality research which allows comparisons between a wide range of interventions
Providers	Professional identity of prehospital critical care provider	Individual/regional All patients treated by provider/EMS	Prove value of own practice and optimise team effectiveness

**Table 3:** Representative quotes from stakeholders regarding their priorities in prehospital research

Stakeholder Group	Quotes (Topic 1: Research Priorities)
PPI group	“So I really would like to think that what comes out of cardiac research is that what I received [prehospital critical care] becomes available nationally.”
Air ambulance charities	“Because we exist through publicly raised funds. […] if something gets out in the media, or it's presented in a way that knocks the charity's credibility or its brand, then, obviously, that could be fatal to these organisations.”
Ambulance service commissioners	“Does [prehospital critical care] give you better outcomes for sufficient numbers of patients to justify that expense?”
Prehospital researchers	“I think it's only worth investing in clinical care if you know that clinical care is effective. And so that's why good research and sound evidence is a prerequisite for good clinical care.”
Prehospital providers	“So I think we continually have to try and prove our worth, don't we. And then if something exposes actually, guys, you're really not needed, well, […] nails in our own coffin.”

### 3.2 Topic 2: randomisation of prehospital critical care

#### 3.2.1 Main themes

- Emotive versus evidence‐based considerations
- Prehospital critical care as a unique health care intervention
- Out‐of‐hospital cardiac arrest as a unique condition

When considering prehospital critical care as a health care intervention for the condition of OHCA, the 5 stakeholder groups came to remarkably different conclusions regarding the ethics of randomising the delivery of such a service within the context of a clinical trial. The reason for this discrepancy can be largely explained through each group's perception of the intervention (prehospital critical care), condition (OHCA), and their understanding of the limitations of observational research. The PPI group considered any method of randomisation to be unethical, while air ambulance charity representatives only accepted scenarios of cluster randomisation or the randomisation of a new drug or intervention which currently was not part of their service. Both groups' arguments against randomisation were based largely on emotive concerns invoked by the imminent life‐or‐death situation of OHCA and the perception of prehospital critical care being beneficial. Withholding a combination of advanced technology (helicopters), interventions (critical care interventions), and expertise (critical care providers) from patients who need these the most would run contrary to the mission statements that most air ambulances advertise: to be there in a time of need and to save lives. Probably the starkest contrast to this point of view was expressed by the group of NHS ambulance service commissioners, who acknowledged the emotive issues of randomisation but were mainly concerned about excessive costs of prehospital critical care. Similarly, the researchers interviewed in this study considered preconceived ideas about benefits or costs or emotive associations as largely irrelevant, unless based on reliable evidence. Finally, for the group of prehospital critical care providers, the intervention of interest was strongly linked to their professional identity and perceived expertise. Randomising prehospital critical care for OHCA was, therefore, seen as ethically difficult but, more importantly, as a gamble that might either support or threaten the providers' professional identity and future role. Table 4 contains representative quotes from all stakeholder groups for this topic.

**Table 4:** Representative quotes from stakeholders regarding randomisation of prehospital critical care for out‐of‐hospital cardiac arrest

Stakeholder Group	Quotes (Topic 2: Randomisation)
PPI group	“[…] they've had a cardiac arrest and they've gone and so the critical care team, someone brings them back to life. And therein lies my main worry. You can never have a true randomised trial. Because you are ultimately playing god.”
Air ambulance charities	“I think it's very, very difficult. [...] I can sort of feel that it's easier with a drug trial to randomise treatment. Because there's no proven benefit to the drug. But when you're saying that some people [pause] would get help or a service, and other people just wouldn't on a random basis, that sounds really bad.”
Ambulance service commissioners	“I would go back to the question, though why would anybody be investing that level of money into [randomising] a critical care team in that way, on a hypothesis which doesn't seem to have much evidence behind it to even get that point?”
Prehospital researchers	“Well whilst [patient‐level randomisation] is not unacceptable to me, I think the best way to do it is probably at a cluster level.”
Prehospital providers	“Basically we're happy to randomise things that we think don't work. Aren't we. And we've got belief that we potentially do [improve outcomes].”

### 3.3 Topic 3: funding decision making

#### 3.3.1 Main themes

- Importance of research for funding decisions
- Impact of factors other than research findings

Given that a definitive randomised controlled trial of prehospital critical care for OHCA is unlikely to be feasible in current circumstances, all stakeholder groups were asked about their views on funding support for this intervention based on limited evidence. Similar to the previous 2 topics, opinions differed significantly between, but not within, the groups. All stakeholder groups acknowledged the importance of research findings in the decision making process. However, each stakeholder group highlighted factors specific to their background, which would also need to be considered and might even outweigh research findings in funding decision making. The PPI group emphasised the need for social acceptability, while charity representatives considered public demand and opinion a significant driver of their funding strategies. Commissioners, on the other hand, acknowledged that budget limitations and policies would potentially limit the impact of research on their decision making process. The stakeholder group of researchers considered factors other than high‐quality evidence to be potentially misleading and, therefore, harmful to patients. Finally, the group of prehospital providers suggested a combination of stakeholder and expert opinion as suitable supplement to observational research in prehospital care. Table 5 contains representative quotes from all stakeholder groups for this topic.

**Table 5:** Representative quotes from stakeholders regarding funding of prehospital critical care for out‐of‐hospital cardiac arrest

Stakeholder Group	Quotes (Topic 3: Funding)
PPI group	“So funding is an emotional decision. […]Which will always be, regardless of whether you have a randomised set of data or observational.”
Air ambulance charities	“What we require in terms of evidence is probably a lot less because we're going to be able to take that view of, well, common sense […]”
Ambulance service commissioners	“That you would look at the strength of evidence but you have to weigh that up against everything else, ie, the cost and what're you going to compromise in terms of other services.”
Prehospital researchers	“So if we're uncertain what to do, then doing more of what's uncertain isn't actually going to improve things for patients. And so I think that's a job for researchers to persuade people that [further] research is needed in those situations.”
Prehospital providers	“[…] this is a very multi‐faceted set of interventions that we're talking about, so once we've got that initial piece of information [research findings], we can drill deeper to find out what is working and what isn't.”

## 4 DISCUSSION

The 5 stakeholder groups in this research displayed divergent views of research and funding strategies in relation to the complex intervention of prehospital critical care for the life‐threatening condition of OHCA. As demonstrated throughout the topics and themes presented in the results section, the reasons for this divergence can largely be explained by the different personal experiences and situational contexts of each stakeholder group. Many aspects of the strategies suggested by the stakeholder groups only partially align with the principles of traditional evidence‐based medicine, but are held with strong conviction. However, despite these often opposing views, a common appreciation of the concepts of scientific enquiry, fairness, and beneficence can be identified, and is further discussed here. Focusing on common values rather than opposing strategies can support successful stakeholder engagement in research of complex interventions.

### 4.1 Fairness and equity

Fairness was referred to frequently by stakeholder groups during the discussion of research priorities and funding decisions, but the interpretation of this concept differed between groups. The PPI group and charity representatives focused strongly on the provision of optimised prehospital care to patients with OHCA or critical illness, respectively. They essentially argued for vertical equity, whereby patients with the greatest need receive the most services.^21^ Out‐of‐hospital cardiac arrest is an unpredictable, and often chaotic and dramatic event, with a 90% mortality rate and potential for psychological distress for survivors and witnesses.^22^, ^23^ Providing a higher level of care for OHCA than for less severe prehospital conditions could be considered a fair approach, when fairness is viewed under the principle of vertical equity.^24^ A limitation to this argument is that vertical equity seeks to balance health resource distribution where outcomes are unnecessarily unfair, rather inevitably unequal.^25^ The question in this context, therefore, becomes one of “*is a 90% mortality rate from OHCA a consequence of insufficient (and unfair) resource distribution? Or is it an inevitable aspect of OHCA, despite adequate treatment?*” The 2 stakeholder groups in this study which argued the latter were the ambulance service commissioners and prehospital researchers. From their perspective, ambulance services already commit a significant amount of resources to the standard treatment of the relatively small number of patients with OHCA.^26^ Further increasing the level of care at increasing costs was perceived as unfair to other patients, in keeping with the principle of horizontal equity.^27^ Patient and public involvement is an increasingly important factor in both research and health care commissioning, and an understanding and awareness of the underlying value judgements of all stakeholders is crucial if these models of shared decision‐making are to prove successful.^28^

### 4.2 Helping patients—the rule of rescue

Probably the most emotionally and ethically challenging aspect of this study was the discussion about randomising prehospital critical care for OHCA, with views ranging from outrage at the very idea (PPI group) to the concept being difficult but acceptable (researchers). While some of this variation can be explained by the different stakeholder perspectives of equipoise and understanding of randomisation, the *Rule of Rescue* also played a role in this debate.^29^ It describes the human instinct to render assistance to individuals in immediate peril, irrespective of the costs and, sometimes, even personal risk. It was the image of experienced health care providers, equipped with advanced technology, standing by, while an identifiable individual was fighting for survival during OHCA, which made the randomisation of individual patients challenging for the majority of stakeholder groups. Removing either the *human expert* element from the intervention or the imminent *life or death* struggle from the condition eased the emotional imperative of the Rule of Rescue during these discussions and made randomisation largely a question of equipoise and logistics. On the other hand, cluster randomisation acts to remove the randomisation process away from the individual and prevents the identification of a single patient in peril, making it the preferred choice of stakeholders in this study. Of note, cluster randomisation of prehospital critical care for OHCA was still considered unethical by the PPI group in this study, and significant ethical challenges, particularly in relation to individual patient rights, remain.^30^ Regarding health care funding decisions, the National Institute for Health and Care Excellent considered the Rule of Rescue in one of its Citizen Reports,^31^ but explicitly did not adopt it in the guidance on social value judgement.^32^

### 4.3 Scientific enquiry

Despite a clearly expressed enthusiasm for, and appreciation of, the importance of prehospital research, views on the relative importance and ideal conduct of this research varied amongst stakeholder groups. The PPI group's views on randomisation of prehospital critical care for OHCA were dominated by a perceived lack of equipoise regarding prehospital critical care and a general unease about the randomisation process during an ongoing OHCA. Dickert et al interviewed survivors of OHCA and their relatives regarding their views on research and randomisation without consent in OHCA.^33^ In contrast to our findings, the OHCA survivors and relatives in Dickert's study were largely supportive of randomisation. This can probably be explained by the fact that the scenarios of randomised controlled trials discussed in Dickert's research involved theoretical simple drug interventions or new, unproven experimental interventions with clear equipoise.^33^ Similarly, participants in our PPI group considered the randomisation of different advanced airway management strategies to be acceptable. Randomisation of prehospital critical care, on the other hand, was deemed unsafe and unethical and was perceived akin to withholding care from patients. In keeping with other previous findings, the PPI group participants perceived the randomisation process as risking trivialisation of significant health care decisions, and did not always appreciate the differences between observational and experimental research designs.^34^, ^35^ The role of charities in UK health care provision is likely to increase over the coming years, providing both opportunities and new challenges for medical research.^36^ To the best of our knowledge, this is the first study describing the views of UK air ambulance charities on their involvement in research. It is encouraging that all charities in this study were supportive of, and involved in, research. However, the restrictions associated with fund raising requirements need to be considered in this complex setting.^37^ Perhaps unsurprisingly, the views of both the commissioners and researchers in this group largely aligned with the principles outlined in National Institute for Health and Care Excellent's *Social Value Judgments* document, namely cost‐effectiveness, fair distribution of resources across the population, and to not offer effective treatment if the costs to the population are inappropriately high.^32^ For the commissioners in our study, these ideals competed with the imperative of achieving externally determined targets and the reality of a limited health care budget.

Finally, our study considered the impact of research on the intervention itself: prehospital critical care providers. Participation in research was seen as consistent with their professional identity as modern and effective health care providers, yet threatened this very identity if negative results ensued. This finding is consistent with previous studies of paramedic involvement in prehospital research, and is an important aspect to consider.^13^, ^38^

### 4.4 Limitations

For logistic reasons, we used a combination of interviews and focus group discussions, and this may have influenced the results. However, the research team actively monitored for any signs of missing participant voices because of the research design, and were satisfied that the exact method of information gathering did not seem to influence the results significantly. While we would have liked to use purposive sampling strategies for all participants, this was only possible for the charity, commissioner, and researcher stakeholder groups. We did not provide participants with a detailed explanation of the counterfactual framework underpinning randomised controlled trials and causal inference. It is possible that such a description would have reduced some of the resistance that participants expressed towards randomisation. However, a full appreciation of these theories is difficult to achieve within a reasonable timeframe and would have altered the perspective of stakeholder groups participating in this research, thus limiting generalisability. Furthermore, the discussions around randomisation were largely driven by emotive or practical considerations, rather than theory. To generalisability, the study's results are based on a relatively small number of participants and we cannot fully exclude the possibility that we did not capture the full extent of views for each stakeholder group. The group of prehospital providers all worked within the same organisation, and might not be representative of other providers working under different circumstances. The PPI group's view was influenced by their personal experience, and it is, therefore, unlikely that they fully represent the general population. The question of representativeness in PPI is a largely unresolved issue.^28^ Our view is that the PPI group in our study is limited in its representation of the general population, but it, nevertheless, represents a major stakeholder in OHCA. Finally, a degree of subjectivity to the data collection process, as well as analysis and presentation of data will have inevitably shaped the results of this research. We, therefore, described the underlying research paradigm in the methods section and placed our findings within the context of existing research in the discussion section. In summary, this exploratory research is by no means an exhaustive representation of all potential stakeholders' views but focuses on important mechanism which determined contrary views of 5 key stakeholder groups.

## 5 CONCLUSION

Analysis of the views of 5 stakeholder groups regarding research and the funding of prehospital critical care for OHCA revealed shared values, but a variety of different strategies to achieve these. The results of this exploratory research can help researchers in similar fields in the planning and presentation of their research, to maximise benefits of stakeholder engagement on decision‐making.

## FUNDING

This work is funded by a National Institute for Health Research (NIHR) doctoral research fellowship for Johannes von Vopelius‐Feldt (DRF‐2015‐08‐040). The funder is not involved in the design of the study or collection, analysis and interpretation of data, or in writing the manuscript. The views expressed are those of the author(s) and not necessarily those of the NHS, the NIHR, or the Department of Health.

## CONFLICT OF INTEREST

Both Jonathan Benger and Johannes von Vopelius‐Feldt work as physicians with the prehospital critical care team of the Great Western Air Ambulance.

## AUTHOR CONTRIBUTIONS

Conceptualization: Johannes von Vopelius‐Feldt, Janet Brandling, Jonathan Benger

Formal Analysis: Johannes von Vopelius‐Feldt, Janet Brandling

Funding Acquisition: Johannes von Vopelius‐Feldt, Janet Brandling, Jonathan Benger

Writing – review and editing: Johannes von Vopelius‐Feldt, Janet Brandling, Jonathan Benger

Writing – original draft: Johannes von Vopelius‐Feldt

## APPENDIX A

**Table A1:** Recruitment strategies for the 5 stakeholder groups of prehospital critical care for out‐of‐hospital cardiac arrest

Stakeholder Group	Recruitment Strategy
Patient and public involvement (PPI) group	All members of a local OHCA research PPI group were invited during 1 of the regular meetings to participate in a focus group discussion.
Representatives of relevant charities	Senior nonclinical members (for example chief executive officers) were contacted by email via the charities' webpages and the Association of Air Ambulances. One‐to‐one interviews were arranged with each participant.
Ambulance commissioners	A group of UK ambulance service commissioners was contacted via email and agreed to arrange a focus group discussion during 1 of the group's regular meetings.
Prehospital researchers	Participants were identified by JVVF through attendance at 2 relevant national conferences, and approached in person or via email. One‐to‐one interviews, either in person or over the phone, were arranged with each participant.
Prehospital providers	A focus group discussion with a group of critical care paramedics (operating in a different ambulance trust to the research team) was set up by JVVF via email.

## APPENDIX B Discussion/interview guide

### B.1 Focus group/interview topic guide (30‐90‐minute session)

#### B.1.1 Title of study

Prehospital critical care for out‐of‐hospital cardiac arrest

Interview objectives

- To identify aims and priorities of research in prehospital care and out‐of‐hospital cardiac arrest (OHCA)
- To understand the values attached to research in OHCA
- To explore how these values are balanced against individual patient rights
- To explore funding decisions in the context of limited evidence

Ground rules (5 minutes)

- Interview/focus group is a relaxed discussion, not a question and answer session
- There are no right or wrong answers
- The aim is to fully explore all views
- The discussion is confidential—comments will not be attributed to anyone
- So please say what you really think and feel
- Support is available from the researcher or his supervisor if distress occurs either during the interview/focus group or later on (contact details on the participant information sheet)
- The interview/discussion will last 30 to 90 minutes

Introductions (5‐10 minutes)

- Researcher introduction
- Summary of the PhD research project: Does prehospital critical care improve survival following OHCA, compared to advanced life support? (Adjust to stakeholder group's background)
- Participant introduction
  - Current role
  - Background

1. General attitudes towards research in prehospital care (15‐20 minutes)
  - Scenario: Imagine being told about a new research study which will recruit patients in prehospital care
    - What are your priorities? Follow‐up questions as needed.
    - Prompt about
      - Ethics
      - Logistics
      - Finances
      - Quality and usefulness of research
      - Reputation
2. Randomised research (15‐20 minutes)
  - Review of evidence levels
    - Case series
    - Observational studies
    - Randomised controlled trials
  - How do you feel about randomisation of prehospital critical care for OHCA?
    - Does this view differ from, for example, randomising a cancer drug?
    - Or a new prehospital technology?
  - Do the participants' views change when considering
    - 1:1 randomisation of existing service
    - 1:n randomisation of existing service
    - 1:1 randomisation of a new service
    - Cluster randomisation of a new service
3. Funding prehospital (critical) care for OHCA (15‐20 minutes)
  - In the absence of definite benefit/cost‐effectiveness, how should funding decisions regarding prehospital critical care for OHCA be made?
    - Consider that not funding also equals a decision
    - What role should observational research play?

Closure (10 minutes)

- Summary of the key points raised
- Do you want to clarify or add anything?
- Is there any other information you think would be useful to share?
- Thank you for participating
